# A new probabilistic model: Theory, simulation and applications to sports and failure times data

**DOI:** 10.1016/j.heliyon.2024.e25651

**Published:** 2024-02-06

**Authors:** Xiangming Tang, Jin-Taek Seong, Randa Alharbi, Aned Al Mutairi, Said G. Nasr

**Affiliations:** a Jiangxi Modern Vocational and Technical College, Nanchang 330000, Jiangxi, China; b Graduate School of Data Science, Chonnam National University, Gwangju 61186, Republic of Korea; c Department of Statistics, Faculty of Science, University of Tabuk, Tabuk, Saudi Arabia; d Department of Mathematical Sciences, College of Science, Princess Nourah bint Abdulrahman University, P.O. Box 84428, Riyadh 11671, Saudi Arabia; e Department of Statistics and Insurance, Faculty of Commerce, Arish University, Al-Arish 45511, Egypt

**Keywords:** Statistical methodology, Flexible Weibull distribution, Estimation, Monte Carlo simulation, Re-injury rate, Engineering data, Statistical modeling

## Abstract

In applied sectors, data modeling/analysis is very important for decision-making and future predictions. Data analysis in applied sectors mainly relies on probability distributions. Data arising from numerous sectors such as engineering-related fields have complex structures. For such kinds of data having complex structures, the implementation of classical distributions is not a suitable choice. Therefore, researchers often need to look for more flexible models that might have the capability of capturing a high degree of kurtosis and increasing the fitting power of the classical models. Taking motivation from the above theory, to achieve these goals, we study a new probabilistic model, which we named a new beta power flexible Weibull (NBPF-Weibull) distribution. We derive some of the main distributional properties of the NBPF-Weibull model. The estimators for the parameters of the NBPF-Weibull distribution are derived. The performances of these estimators are judged by incorporating a simulation study for different selected values of the parameters. Three data sets are used to demonstrate the applicability of the NBPF-Weibull model. The first data set is observed from sports. It represents the re-injury rate of various football players. While the other two data sets are observed from the reliability zone. By adopting certain diagnostic criteria, it is proven that the NBPF-Weibull model repeatedly surpasses well-known classical and modified models.

## 1 Introduction

Assessing the multiform of data is very important to achieve more accurate and precise decisions. Selecting an appropriate model for capturing the complex form of data is very crucial. Over recent years, numerous probability models have been suggested to fit data sets with different behaviors and shapes. However, adopting a classical distribution to fit complex data sets can lead to unreliable results. Hence, there is clearly evidence for researchers to modify the classical models to enhance their fitting capability [1], [2], [3], [4], [5], [6], [7], [8], [9], [10], [11].

In the group of existing probabilistic models (for instance, introduced, studied, and implemented models), the Weibull distribution has gained considerable attention. The Weibull distribution has certain advantages over the other classical distributions because of its different monotonic hazard shapes and closed-form distribution function. For reliability and healthcare data analyses, most often the utilization/employment of the Weibull model is considered [12], [13], [14], [15], [16], [17], [18], [19].

Assume *W* has the Weibull model expressed by W∼Wei(α,δ), if its CDF (cumulative distribution function) G(w;α,δ) is

(1) $$G\left(w;\alpha ,\delta \right)=1-e^{-\delta w^{\alpha }},\;\;\;\;\;\;\;w\geq 0,$$

where *α* and δ>0. The related hazard function (HF) hG(w;α,δ) is

$$h\left(w;\alpha ,\delta \right)=\alpha \delta w^{\alpha -1},\;\;\;\;\;\;\;w>0.$$

**Figure 1:**
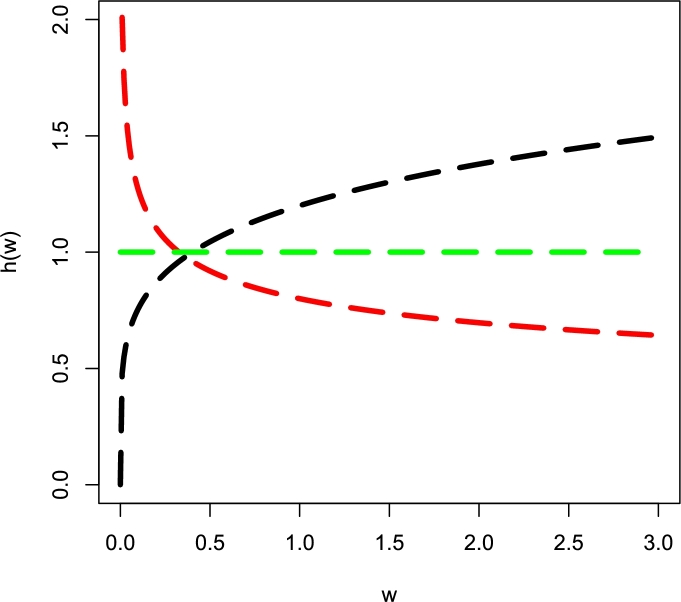
The visual illustrations of h(w;α,δ) for α=(0.8,1,1.2) and δ=(1,1,1).

Some illustrations of h(w;α,δ) for (*i*) δ=1,α=0.8 (red-color line), (*ii*) δ=1,α=1 (green-color line), and (*iii*) δ=1,α=1.2 (black-color line) are presented in Fig. 1.

Fig. 1 shows three possible behaviors of h(w;α,δ) such as (*i*) increasing (α>1), (*i*) decreasing (α<1), or (*i*) constant (α=1) shape.

From Fig. 1, it is obvious that when data sets have complex hazard shapes (i.e., non-monotonic), the Weibull model may not be an optimal model for these types of data sets. To overcome this weakness of the Weibull model, its several flexible extensions have been proposed. One of the most interesting modifications of Eq. (1) is called flexible Weibull (F-Weibull) distribution. Its CDF is

(2) $$G\left(w;\alpha ,\delta \right)=1-e^{-e^{\left(\delta w-\frac{\alpha }{w}\right)}},\;\;\;\;\;\;\;w\geq 0,$$

where α>0 and δ>0. With linked to Eq. (2), the associated PDF (probability density function) is

(3) $$G\left(w;\alpha ,\delta \right)=\left(\delta +\frac{\alpha }{w^{2}}\right)e^{\left(\delta w-\frac{\alpha }{w}\right)}e^{-e^{\left(\delta w-\frac{\alpha }{w}\right)}},\;\;\;\;\;\;\;w>0.$$

The F-Weibull model is furthermore extended and studied by (ii) El-Gohary et al. [20] introducing the inverse form of the F-Weibull model, (iii) El-Damcese et al. [21] introducing the Kumaraswamy version of the F-Weibull model, and (iv) El-Morshedy et al. [22] proposing the exponentiated inverse form of the F-Weibull model.

This study also provides a improvised version of Eq. (2) by implementing the beta power transformed method [23]. For W∈R the CDF of the new beta power transformed method is

(4) $$F\left(w;\beta ,\Xi \right)=\frac{\beta^{G\left(w;\Xi \right)}-\left[1-G\left(w;\Xi \right)\right]}{\beta },$$

with PDF

$$f\left(w;\beta ,\Xi \right)=\frac{g\left(w;\Xi \right)}{\beta }\left[1+\left(\log\beta \right)G\left(w;\Xi \right)\right],$$

where **Ξ** is a parameter vector linked with G(w;Ξ). It is important to keep in view that when β=1, then Eq. (4) reduces G(w;Ξ).

In the very next section, we use Eq. (2) along with Eq. (4) to study another improvised extension of the F-Weibull distribution. The improvised probability model is called a NBPF-Weibull distribution. Some visual displays of its PDF and hazard function (HF) are also presented. Section 3 provides the mathematical description of the estimators of the NBPF-Weibull model. The estimators of the NBPF-Weibull model are mathematically derived in Section 3. Furthermore, the simulation study for different values of *α*, *δ*, and *β* is also conducted in Section 3. Section 4 provides three practical examples. The concluding overview, demerits of this study, and future research plans are discussed in Section 5.

## 2 The NBPF-Weibull: model description

Let *W* have the F-Weibull model with CDF G(w;α,δ) and PDF g(w;α,δ) presented in Eq. (2) and Eq. (3), respectively. Then, using Eq. (2) in Eq. (4), we get

(5) $$F\left(w;\beta ,\Xi \right)=\frac{\beta^{1-e^{-e^{\left(\delta w-\frac{\alpha }{w}\right)}}}-e^{-e^{\left(\delta w-\frac{\alpha }{w}\right)}}}{\beta },\;\;\;\;\;\;\;w>0,$$

where Ξ=(δ,α). Clearly, for β=1, Eq. (5) reduces to Eq. (2). The survival function (SF) is

$$S\left(w;\beta ,\Xi \right)=\frac{\beta -\beta^{1-e^{-e^{\left(\delta w-\frac{\alpha }{w}\right)}}}+e^{-e^{\left(\delta w-\frac{\alpha }{w}\right)}}}{\beta },\;\;\;\;\;\;\;w>0.$$

**Figure 2:**
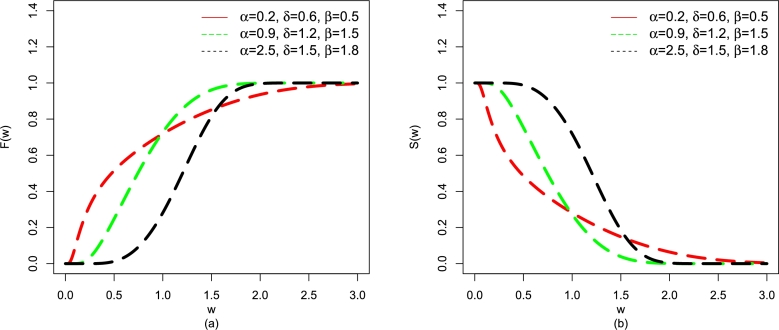
Plots of (a) F(w;β,Ξ) and (b) S(w;β,Ξ) for α=(0.2,0.9,2.5), δ=(0.6,1.2,1.5), and β=(0.5,1.5,1.8).

For α=(0.2,0.9,2.5), δ=(0.6,1.2,1.5), and β=(0.5,1.5,1.8), the plots for F(w;β,Ξ) and S(w;β,Ξ) are given in Fig. 2(a-b).

Furthermore, in link to Eq. (5), the PDF is

(6) $$f\left(w;\beta ,\Xi \right)=\frac{\left(\delta +\frac{\alpha }{w^{2}}\right)e^{\left(\delta w-\frac{\alpha }{w}\right)}e^{-e^{\left(\delta w-\frac{\alpha }{w}\right)}}}{\beta }\left[\left(\log\beta \right)\left(1-e^{-e^{\left(\delta w-\frac{\alpha }{w}\right)}}\right)+1\right],\;\;w>0.$$

In link to Eq. (5) and Eq. (6), the HF and cumulative HF are given by

$$h\left(w;\beta ,\Xi \right)=\frac{\left(\delta +\frac{\alpha }{w^{2}}\right)e^{\left(\delta w-\frac{\alpha }{w}\right)}e^{-e^{\left(\delta w-\frac{\alpha }{w}\right)}}}{\beta -\beta^{1-e^{-e^{\left(\delta w-\frac{\alpha }{w}\right)}}}+e^{-e^{\left(\delta w-\frac{\alpha }{w}\right)}}}\left[\left(\log\beta \right)\left(1-e^{-e^{\left(\delta w-\frac{\alpha }{w}\right)}}\right)+1\right],\;\;w>0,$$

and

$$H\left(w;\beta ,\Xi \right)=-\log\left(\frac{\beta -\beta^{1-e^{-e^{\left(\delta w-\frac{\alpha }{w}\right)}}}+e^{-e^{\left(\delta w-\frac{\alpha }{w}\right)}}}{\beta }\right),\;\;\;\;\;\;\;w>0,$$

respectively.

**Figure 3:**
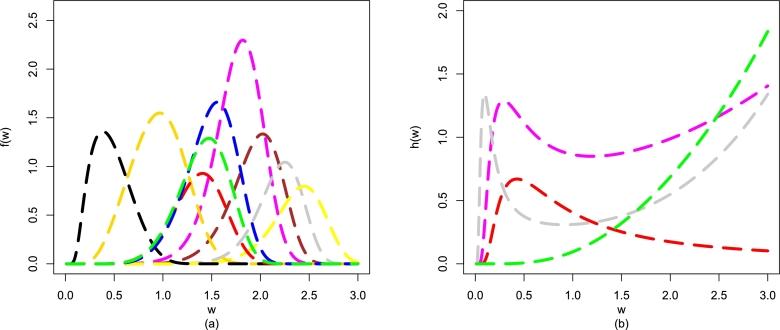
Plots of (a) f(w;β,Ξ) and (b) h(w;β,Ξ) of the proposed model.

Some plots of f(w;β,Ξ) as well as the plots of h(w;β,Ξ) are sketched in Fig. 3(a-b). The plots of f(w;β,Ξ) are obtained for α=5.5,δ=1.8,β=0.4 (magenta-color line), α=9.5,δ=2.2,β=1.4 (brown-color line), α=13.5,δ=2.5,β=2.5 (grey-color line), α=14.2,δ=2.2,β=3.1 (yellow-color line), α=1.9,δ=1.8,β=0.8 (gold-color line), α=0.7,δ=2.2,β=1.9 (black-color line), α=4.8,δ=2.1,β=2.4 (red-color line), α=5.3,δ=2.1,β=0.9 (blue-color line), and α=5.0,δ=2.1,β=1.4 (green-color line). Fig. 3(a-b) shows f(w;β,Ξ) that is given in Eq. (6) has different shapes. From Fig. 3(a-b), it is clear that *β* has a significant effect on the shapes of f(w;β,Ξ).

In addition, different illustrations of h(w;β,Ξ) also are given in Fig. 3(a-b). The illustrations of h(w;β,Ξ) are sketched for α=0.5,δ=0.4,β=0.9 (magenta-color line), α=0.8,δ=0.02,β=0.7 (red-color line), α=0.2,δ=0.9,β=5.2 (grey-color line), and α=3.1,δ=0.9,β=0.4 (green-color line). These illustrations show that h(w;β,Ξ) has (*i*) increasing (*ii*) unimodal, or (*iii*) modified unimodal shapes.

## 3 Estimation and simulation

This section is reserved for carrying out two objectives. The first objective is to obtain the maximum likelihood estimators (MLEs) $\left({\overset{ˆ}{\alpha }}_{MLE},{\overset{ˆ}{\delta }}_{MLE},{\overset{ˆ}{\beta }}_{MLE}\right)$ for (α,δ,β) of the NBPF-Weibull model. Second, it provides a simulation study to see the behaviors of ${\overset{ˆ}{\alpha }}_{MLE},{\overset{ˆ}{\delta }}_{MLE}$, and ${\overset{ˆ}{\beta }}_{MLE}$.

### 3.1 Estimation

Let a set of randomly observed samples, denoted by $W_{1},W_{2},...,W_{n}$, are taken from the NBPF-Weibull distribution. The associated likelihood function (LF) $\lambda \left(\beta ,\Xi |w_{1},w_{2},...,w_{n}\right)$ is given by

$$\lambda \left(\beta ,\Xi |w_{1},w_{2},...,w_{n}\right)=\prod_{i=1}^{n}\frac{\left(\delta +\frac{\alpha }{w_{i}^{2}}\right)A_{i}\left(w\right)e^{-A_{i}\left(w\right)}}{\beta }\times \left[\left(\log\beta \right)\left(1-e^{-A_{i}\left(w\right)}\right)+1\right].$$

Corresponding to $\lambda \left(\beta ,\Xi |w_{1},w_{2},...,w_{n}\right)$, the log LF $\lambda \left(w_{1},w_{2},...,w_{n}|\beta ,\Xi \right)$ is given by

$$\lambda \left(w_{1},w_{2},...,w_{n}|\beta ,\Xi \right)=\sum_{i=1}^{n}\log\left(\delta +\frac{\alpha }{w_{i}^{2}}\right)+\sum_{i=1}^{n}\left(\delta w_{i}-\frac{\alpha }{w_{i}}\right)-\sum_{i=1}^{n}A_{i}\left(w\right)-n\log\beta +\sum_{i=1}^{n}\log\left[\left(\log\beta \right)\left(1-e^{-A_{i}\left(w\right)}\right)+1\right].$$

The derivatives of $\lambda \left(w_{1},w_{2},...,w_{n}|\beta ,\Xi \right)$ are given by

$$\frac{\partial }{\partial \alpha }\lambda \left(w_{1},w_{2},...,w_{n}|\beta ,\Xi \right)=\sum_{i=1}^{n}\frac{\frac{1}{w_{i}^{2}}}{\left(\delta +\frac{\alpha }{w_{i}^{2}}\right)}-\sum_{i=1}^{n}\frac{1}{w_{i}}+\sum_{i=1}^{n}\frac{1}{w_{i}}e^{\left(\delta w_{i}-\frac{\alpha }{w_{i}}\right)}-\left(\log\beta \right)\sum_{i=1}^{n}\frac{\frac{1}{w_{i}}A_{i}\left(w\right)e^{-A_{i}\left(w\right)}}{\left[\left(\log\beta \right)\left(1-e^{-A_{i}\left(w\right)}\right)+1\right]},$$

$$\frac{\partial }{\partial \delta }\lambda \left(w_{1},w_{2},...,w_{n}|\beta ,\Xi \right)=\sum_{i=1}^{n}\frac{1}{\left(\delta +\frac{\alpha }{w_{i}^{2}}\right)}-\sum_{i=1}^{n}w_{i}-\sum_{i=1}^{n}w_{i}A_{i}\left(w\right)+\left(\log\beta \right)\sum_{i=1}^{n}\frac{w_{i}A_{i}\left(w\right)e^{-A_{i}\left(w\right)}}{\left[\left(\log\beta \right)\left(1-e^{-A_{i}\left(w\right)}\right)+1\right]},$$

and

$$\frac{\partial }{\partial \beta }\lambda \left(w_{1},w_{2},...,w_{n}|\beta ,\Xi \right)=-\frac{n}{\beta }+\sum_{i=1}^{n}\frac{\frac{1}{\beta }\left(1-e^{-A_{i}\left(w\right)}\right)}{\left[\left(\log\beta \right)\left(1-e^{-A_{i}\left(w\right)}\right)+1\right]},$$

where $A_{i}\left(w\right)$.

By solving the expressions $\frac{\partial }{\partial \alpha }\lambda \left(w_{1},w_{2},...,w_{n}|\beta ,\Xi \right)=0,\frac{\partial }{\partial \delta }\lambda \left(w_{1},w_{2},...,w_{n}|\beta ,\Xi \right)=0$, and $\frac{\partial }{\partial \beta }\lambda \left(w_{1},w_{2},...,w_{n}|\beta ,\Xi \right)=0$, we obtain ${\overset{ˆ}{\alpha }}_{MLE},{\overset{ˆ}{\delta }}_{MLE}$, and ${\overset{ˆ}{\beta }}_{MLE}$, respectively.

### 3.2 Simulation

Here, we cover the second aim of this section by carrying out a simulation study. For this purpose, we generate random numbers from the NBPF-Weibull distribution. Random numbers from the NBPF-Weibull distribution of sizes, say, n=100,200,300,...,1000 are generated. The simulation study of the NBPF-Weibull distribution is conducted for three sets of α,δ, and *β*.

We choose two evaluation criteria and implement them for checking the performances of ${\overset{ˆ}{\alpha }}_{MLE},{\overset{ˆ}{\delta }}_{MLE}$, and ${\overset{ˆ}{\beta }}_{MLE}$. These criteria are

$$MSE\left({\overset{ˆ}{\tau }}_{MLE}\right)=\frac{1}{1000}\sum_{i=1}^{1000}{\left({\overset{ˆ}{\tau }}_{i}-\tau \right)}^{2},$$

and

$$Bias\left({\overset{ˆ}{\tau }}_{MLE}\right)=\frac{1}{1000}\sum_{i=1}^{1000}\left({\overset{ˆ}{\tau }}_{i}-\tau \right),$$

where τ=(α,δ,β).

For the NBPF-Weibull model, the simulation study is conducted for

• α=1.7,δ=0.9,β=1.5,
• α=1.5,δ=0.6,β=1.1, and
• α=1.9,δ=0.4,β=1.7.

It is a known fact that simulation studies are based on default values (or predefined values) of parameters. As we have mentioned the range of $\alpha \left(\alpha \in R^{+}\right)$, $\delta \left(\delta \in R^{+}\right)$, and $\beta \left(\beta \in R^{+}\right)$. So, within their given ranges, we can choose any value of α,δ, and *β* to carry out the simulation study.

**Figure 4:**
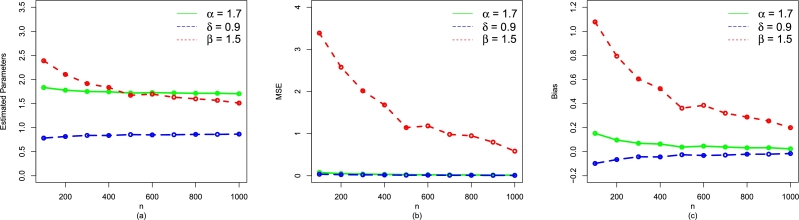
A visual representation of the performances of (a) Estimated Parameters (b) MSE, and (c) Bias of the NBPF-Weibull distribution for *α* = 1.7,*δ* = 0.9, and *β* = 1.5.

**Figure 5:**
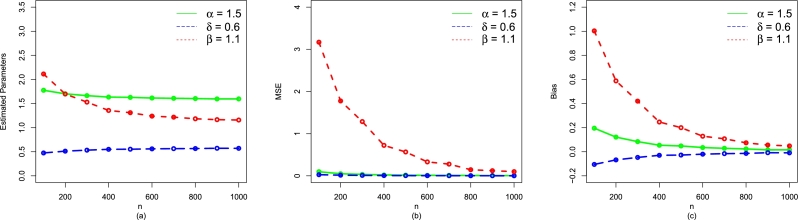
A visual representation of the performances of (a) Estimated Parameters (b) MSE, and (c) Bias of the NBPF-Weibull distribution for *α* = 1.5,*δ* = 0.6, and *β* = 1.1.

**Figure 6:**
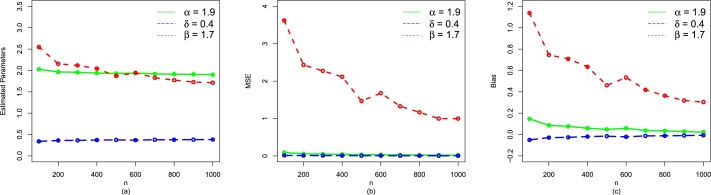
A visual representation of the performances of (a) Estimated Parameters (b) MSE, and (c) Bias of the NBPF-Weibull distribution for *α* = 1.9,*δ* = 0.4, and *β* = 1.7.

The simulation result for the parameter values α=1.7,δ=0.9, and β=1.5 are given in Table 1 (numerical illustration) and Fig. 4(a-c) (visual illustration). For the second set (i.e., α=1.5,δ=0.6,β=1.1), Table 2 and Fig. 5(a-c) provide the numerical and visual illustration of the simulation results, respectively. Whereas, the simulation results for the third set are (i.e., α=1.9,δ=0.4,β=1.7) presented in Table 3 and Fig. 6(a-c). We adopted the well-known R-script with L-BFGS-B method for obtaining the simulation results that are given by Table 1, Table 2, Table 3. From Fig. 4(a-c), Fig. 5(a-c), and Fig. 6(a-c) as well as Table 1, Table 2, Table 3, we can see that as the size of *n* increases, the

• MSEs of ${\overset{ˆ}{\alpha }}_{MLE},{\overset{ˆ}{\delta }}_{MLE}$, and ${\overset{ˆ}{\beta }}_{MLE}$ decay to zero.
• Biases of ${\overset{ˆ}{\alpha }}_{MLE},{\overset{ˆ}{\delta }}_{MLE}$, and ${\overset{ˆ}{\beta }}_{MLE}$ decreases.
• MLEs of α,δ, and *β* decreases.

**Table 1:** The simulation results for *α* = 1.7,*δ* = 0.9, and *β* = 1.5.

*n*	Parameters	MLEs	MSEs	Biases
100	*α*	1.8273340	0.07898099	0.14733363
	*δ*	0.7883497	0.03602651	-0.09165028
	*β*	2.3832950	3.43368930	1.07329470

200	*α*	1.7878860	0.05287926	0.10788643
	*δ*	0.8026612	0.02792825	-0.07733877
	*β*	2.1695750	2.80015690	0.85957470

300	*α*	1.7499220	0.03707273	0.06992165
	*δ*	0.8325429	0.02070612	-0.04745714
	*β*	1.8681920	1.73884120	0.55819240

400	*α*	1.7356180	0.03036621	0.05561794
	*δ*	0.8429907	0.01700806	-0.03700926
	*β*	1.7690010	1.45009400	0.45900140

500	*α*	1.7242040	0.02480372	0.04420408
	*δ*	0.8501755	0.01421308	-0.02982448
	*β*	1.6926850	1.21273380	0.38268500

600	*α*	1.7283040	0.02377011	0.04830412
	*δ*	0.8490129	0.01368064	-0.03098713
	*β*	1.7000390	0.45378161	0.39003910

700	*α*	1.7174430	0.02171580	0.03744342
	*δ*	0.8547041	0.01270919	-0.02529588
	*β*	1.6482280	1.08201710	0.33822790

800	*α*	1.7171150	0.01766425	0.03711469
	*δ*	0.8555187	0.01033979	-0.02448132
	*β*	1.5866320	0.86471860	0.27663220

900	*α*	1.7112450	0.01630920	0.03124496
	*δ*	0.8573766	0.00981596	-0.02262337
	*β*	1.5801540	0.82717680	0.27015350

1000	*α*	1.7103880	0.01439024	0.03038813
	*δ*	0.8988609	0.00863651	-0.02113908
	*β*	1.5107370	0.67976710	0.23073730

**Table 2:** The simulation results for *α* = 1.5,*δ* = 0.6, and *β* = 1.1.

*n*	Parameters	MLEs	MSEs	Biases
100	*α*	1.7968940	0.11016393	0.21689427
	*δ*	0.4630926	0.03005662	-0.11690738
	*β*	2.2036730	3.47403478	1.02367334

200	*α*	1.7045830	0.05520722	0.12458297
	*δ*	0.5111483	0.01677864	-0.06885167
	*β*	1.7456570	1.99974720	0.63565690

300	*α*	1.6613250	0.03477951	0.08132536
	*δ*	0.5344094	0.01111800	-0.04559058
	*β*	1.4948240	1.15347913	0.38482362

400	*α*	1.6360640	0.02192804	0.05606384
	*δ*	0.5478031	0.00746546	-0.03219691
	*β*	1.3677810	0.74050768	0.25778133

500	*α*	1.6324990	0.01959787	0.05249861
	*δ*	0.5497378	0.00693443	-0.03026221
	*β*	1.3504760	0.70033367	0.24047597

600	*α*	1.6206820	0.01554066	0.04068197
	*δ*	0.5565345	0.00527246	-0.02346549
	*β*	1.2874600	0.50807830	0.17746007

700	*α*	1.6085900	0.01050066	0.02858985
	*δ*	0.5649577	0.00369808	-0.01504225
	*β*	1.2252430	0.29091822	0.11524267

800	*α*	1.5560650	0.00704030	0.02606507
	*δ*	0.5659488	0.00261742	-0.01405115
	*β*	1.1730410	0.09965406	0.06304104

900	*α*	1.5372780	0.00603093	0.01727767
	*δ*	0.5804222	0.00226018	-0.00957783
	*β*	1.1570760	0.06806511	0.04707629

1000	*α*	1.5161980	0.00585596	0.01619756
	*δ*	0.5911531	0.00205210	-0.00884689
	*β*	1.1252600	0.14343205	0.05526016

**Table 3:** The simulation results for *α* = 1.9,*δ* = 0.4, and *β* = 1.7.

*n*	Parameters	MLEs	MSEs	Biases
100	*α*	2.0334890	0.09084001	0.15348907
	*δ*	0.3370644	0.01315943	-0.05293558
	*β*	2.6038280	3.62860600	1.13382750

200	*α*	1.9642800	0.05361141	0.08427969
	*δ*	0.3626675	0.00941822	-0.02733246
	*β*	2.1875950	2.61349700	0.77759460

300	*α*	1.9432480	0.04616048	0.06324762
	*δ*	0.3690945	0.00861864	-0.02090545
	*β*	2.0824240	2.27598600	0.67242410

400	*α*	1.9414490	0.03958648	0.06144921
	*δ*	0.3693644	0.00758026	-0.02063564
	*β*	2.0137757	2.00049600	0.60377540

500	*α*	1.9322490	0.03458542	0.05224906
	*δ*	0.3716685	0.00678757	-0.01833154
	*β*	1.9656700	1.79815600	0.55567030

600	*α*	1.9222120	0.02896691	0.04221231
	*δ*	0.3755684	0.00596314	-0.01443156
	*β*	1.9017700	1.66614100	0.49177030

700	*α*	1.9241050	0.02807300	0.04410464
	*δ*	0.3750122	0.00561178	-0.01498775
	*β*	1.8521680	1.41099900	0.44216790

800	*α*	1.9131040	0.02504952	0.03310368
	*δ*	0.3792192	0.00504685	-0.01078080
	*β*	1.7711300	1.13867800	0.36112990

900	*α*	1.9162390	0.02171132	0.03623876
	*δ*	0.3771225	0.00444450	-0.01287746
	*β*	1.7717320	1.12779500	0.36173150

1000	*α*	1.9114440	0.02011196	0.03144390
	*δ*	0.3985021	0.00405697	-0.01149794
	*β*	1.7154500	1.02423200	0.32544970

## 4 Applications

Here, we fit the NBPF-Weibull model to three practical data sets, aiming to show the usefulness of the NBPF-Weibull distribution in applied sectors. These datasets are taken from sports and different fields of engineering.

### 4.1 Data sets

This subsection considers the numerical and visual evaluation of the sports and engineering data sets.

#### 4.1.1 Sports data

The first data set (we set out to represent it with Data 1) is selected from sports sciences, specifically, it is observed from various football matches. It represents the rate of the re-injury of various players in different football matches [24].

**Figure 7:**
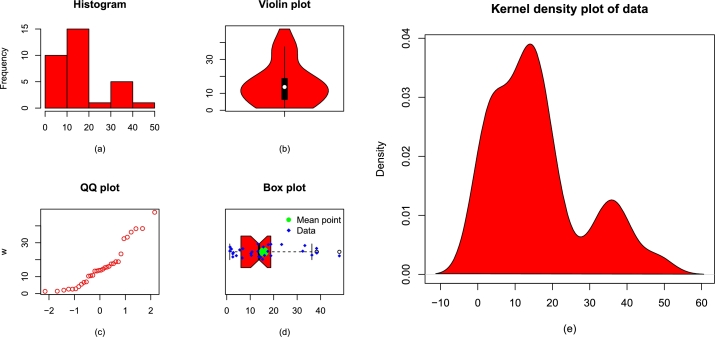
Visual illustrations of Data 1 using (a) display of histogram, (b) violin plot, (c) display of QQ plot, (d) box plot, and (e) kernel density estimator.

The key measures of the re-injury rate of the football's players are: the smallest value = 1.300, maximum value = 48.000, $Q_{1}$ = 6.325, median = 13.750, $Q_{3}$ = 18.800, mean = 15.806, skewness = 0.944, kurtosis = 3.083, variance = 154.100, and range = 46.700. For the sake of simplicity, we represent the re-injury data by Data 1. Fig. 7(a-e) illustrates some basic plots of Data 1.

#### 4.1.2 Reliability data

**Figure 8:**
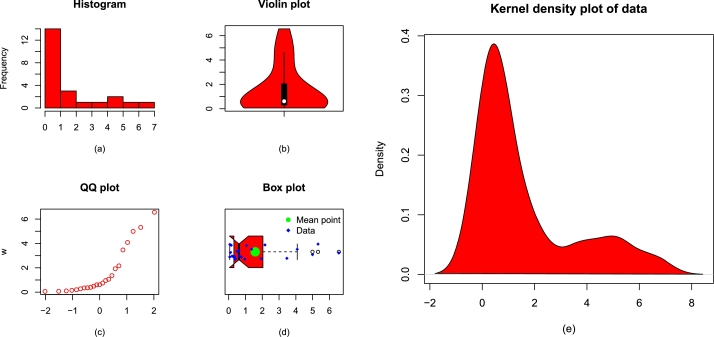
Visual illustrations of Data 2 using (a) display of histogram, (b) violin plot, (c) display of QQ plot, (d) box plot, and (e) kernel density estimator.

The second data set (we set out to represent it with Data 2) shows the failure time of the electronic reactor pumps. More detail about this data can be found in [25]. The waiting time is measured in hours. The key measures of the waiting time data are: the smallest value = 0.062, maximum value = 6.560, $Q_{1}$ = 0.310, median = 0.614, $Q_{3}$ = 2.041, mean = 1.578, skewness = 1.364345, kurtosis = 3.544534, variance = 3.727516, and range = 6.498. For the sake of simplicity, we represent it with Data 2. Fig. 8(a-e) illustrates some basic plots of Data 2.

**Figure 9:**
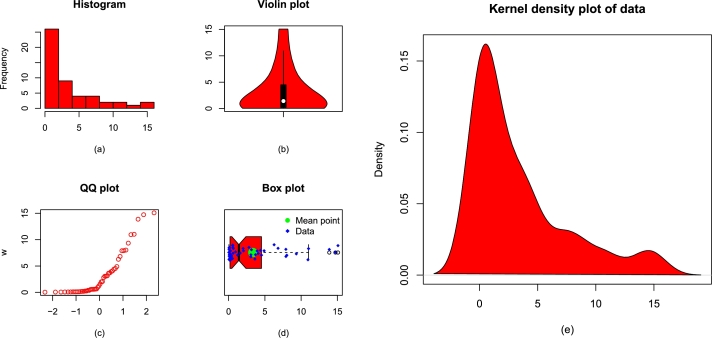
Visual illustrations of Data 3 using (a) display of histogram, (b) violin plot, (c) display of QQ plot, (d) box plot, and (e) kernel density estimator.

The third data (we set out to represent it with Data 3) shows the failure times (per 1000 h) of electronic machines consisting of fifty components [26]. The basic measures of the failure times data (i.e., Data 3) are: the smallest value = 0.0360, maximum value = 15.0800, $Q_{1}$ = 0.2075, median = 1.4140, $Q_{3}$ = 4.4988, mean = 3.3430, skewness = 1.416739, kurtosis = 4.084622, variance = 17.48477, and range = 15.044. Fig. 9(a-e) illustrates some basic plots of Data 3.

### 4.2 Competing distributions

We accomplish the superiority of the NBPF-Weibull distribution over the Weibull distribution and F-Weibull distribution. We also compare its fitting results with other modifications of the F-Weibull and Weibull distributions, namely, exponentiated F-Weibull (EF-Weibull) and exponentiated Weibull (E-Weibull) distributions, respectively. The SFs of the selected models (mentioned above) are given by

• Weibull distribution [27]
  $$S\left(w\right)=e^{-\delta w^{\alpha }},\;\;\;\;\;\;\;\alpha ,\delta >0.$$
• E-Weibull distribution [28]
  $$S\left(w\right)=1-{\left(1-e^{-\delta w^{\alpha }}\right)}^{\gamma },\;\;\;\;\;\;\;\alpha ,\delta ,\gamma >0.$$
• F-Weibull distribution [29]
  $$S\left(w\right)=e^{-e^{\left(\delta w-\frac{\alpha }{w}\right)}},\;\;\;\;\;\;\;\alpha ,\delta >0.$$
• EF-Weibull distribution [30]
  $$S\left(w\right)=1-{\left(1-e^{-e^{\left(\delta w-\frac{\alpha }{w}\right)}}\right)}^{\gamma },\;\;\;\;\;\;\;\alpha ,\delta ,\gamma >0.$$

### 4.3 Information criteria

The comparison between NBPF-Weibull and the above-selected models is made using well-known statistical approaches (i.e., certain information criteria). The information criteria (IC) chosen as comparative tools are obtained as follows

• Akaike IC (AIC)
  2m−2λ.
• Bayesian IC (BIC)
  mlog⁡(n)−2λ.
• Consistent Akaike IC (CAIC)
  $$\frac{2nm}{n-m-1}-2\lambda .$$
• Hannan Quinn IC (HQIC)
  2mlog⁡[log⁡(n)]−2λ.

In these criteria, *m* represents the parameters of the probability distribution applied to the data, *n* represents the number of observations of the underlined data, and *λ* represents the LLF. These criteria are widely implemented to discriminate the most appropriate and optimal model among the rival models. The values of the IC are computed using the R-script with SANN method. Furthermore, the empirical plots of the NBPF-Weibull distribution are also obtained using the R-script.

### 4.4 Analysis of Data 1

**Figure 10:**
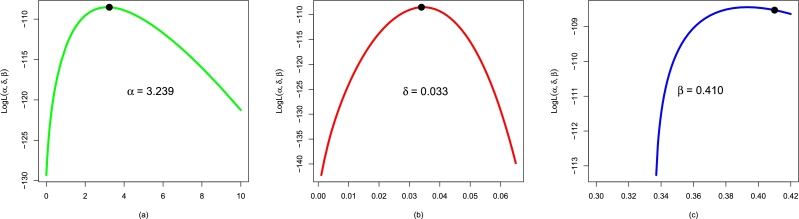
Using Data 1, the profiles plots of (a) ${\overset{ˆ}{\alpha }}_{MLE}$, (b) ${\overset{ˆ}{\delta }}_{MLE}$, and (c) ${\overset{ˆ}{\beta }}_{MLE}$ of the NBPF-Weibull distribution.

This subsection considers the performances of the applied distributions using the re-injury rate (or Data 1). We apply the NBPF-Weibull and all the above-competing distributions to Data 1. The values of ${\overset{ˆ}{\alpha }}_{MLE},{\overset{ˆ}{\delta }}_{MLE},{\overset{ˆ}{\beta }}_{MLE}$, and ${\overset{ˆ}{\gamma }}_{MLE}$ are presented in Table 4. Furthermore, Fig. 10(a-c) illustrates the plots for the profiles of ${\overset{ˆ}{\alpha }}_{MLE}$, ${\overset{ˆ}{\delta }}_{MLE}$, and ${\overset{ˆ}{\beta }}_{MLE}$ of the NBPF-Weibull distribution.

**Table 4:** For Data 1, the values of ${\overset{ˆ}{\alpha }}_{MLE},{\overset{ˆ}{\delta }}_{MLE},{\overset{ˆ}{\beta }}_{MLE}$, and ${\overset{ˆ}{\gamma }}_{MLE}$.

Model	${\overset{ˆ}{\alpha }}_{MLE}$	${\overset{ˆ}{\delta }}_{MLE}$	${\overset{ˆ}{\beta }}_{MLE}$	${\overset{ˆ}{\gamma }}_{MLE}$
NBPF-Weibull	3.239	0.033	0.410	-
Weibull	1.283	0.026	-	-
F-Weibull	5.855	0.031	-	-
EF-Weibull	1.774	0.036	-	2.603
E-Weibull	1.313	0.019	-	0.849

Using Data 1, Table 5 provides the IC values of the NBPF-Weibull distribution and its related competing distributions. For the NBPF-Weibull model, we have AIC = 223.057, CAIC = 223.915, BIC = 227.455, and HQIC = 224.515; see Table 5. Thus, Table 5 categorically confirms that the NBPF-Weibull distribution has the lowest IC values for Data 1. Therefore, it is obvious like a crystal that the NBPF-Weibull distribution can be ranked as the $1^{st}$ as it provides a closer fit to Data 1.

**Table 5:** The criterion values of the fitted models using Data 1.

Model	AIC	CAIC	BIC	HQIC
NBPF-Weibull	223.057	223.915	227.455	224.515
Weibull	242.016	242.430	244.948	242.988
F-Weibull	243.537	243.951	246.469	244.509
EF-Weibull	241.273	242.130	245.670	242.731
E-Weibull	244.350	245.207	248.747	245.808

**Figure 11:**
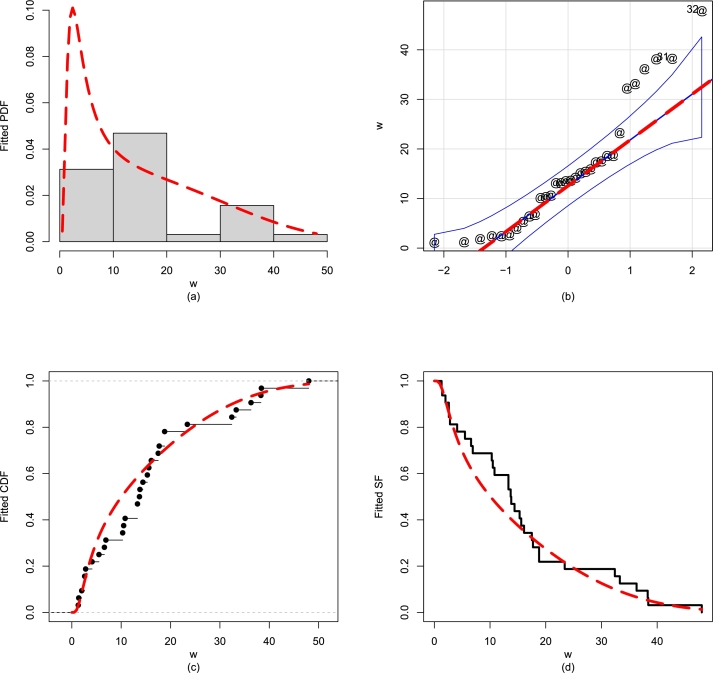
For Data 1, the illustrations of the (a) fitted PDF, (b) QQ plot, (c) empirical CDF, and (d) fitted SF of the NBPF-Weibull distribution.

For Data 1, we also show the performance of the NBPF-Weibull distribution graphically. For visually illustrating the fitted distributions, we select the QQ (quantile-quantile), empirical PDF, SF, PP (probability probability), and estimated CDF; see Fig. 11(a-d). The graphical checking tools also support the close fitting ability of the NBPF-Weibull distribution.

### 4.5 Analysis of Data 2

**Figure 12:**
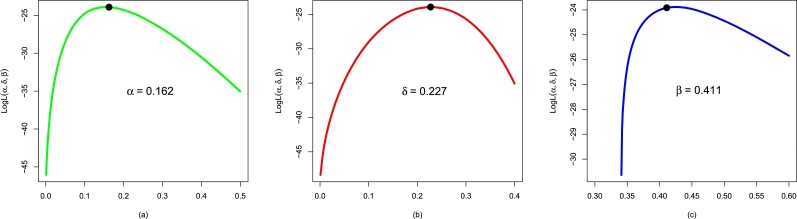
Using Data 2, the profiles plots of (a) ${\overset{ˆ}{\alpha }}_{MLE}$, (b) ${\overset{ˆ}{\delta }}_{MLE}$, and (c) ${\overset{ˆ}{\beta }}_{MLE}$ of the NBPF-Weibull distribution.

Here, we show the performances of the applied distributions using the electronic reactor pumps data (i.e., Data 2) We apply the NBPF-Weibull and all the above-competing distributions to Data 2. The values of ${\overset{ˆ}{\alpha }}_{MLE},{\overset{ˆ}{\delta }}_{MLE},{\overset{ˆ}{\beta }}_{MLE}$, and ${\overset{ˆ}{\gamma }}_{MLE}$ are presented in Table 6. Furthermore, Fig. 12(a-c) illustrates the plots for the profiles of ${\overset{ˆ}{\alpha }}_{MLE}$, ${\overset{ˆ}{\delta }}_{MLE}$, and ${\overset{ˆ}{\beta }}_{MLE}$ of the NBPF-Weibull distribution.

**Table 6:** For Data 2, the values of ${\overset{ˆ}{\alpha }}_{MLE},{\overset{ˆ}{\delta }}_{MLE},{\overset{ˆ}{\beta }}_{MLE}$, and ${\overset{ˆ}{\gamma }}_{MLE}$.

Model	${\overset{ˆ}{\alpha }}_{MLE}$	${\overset{ˆ}{\delta }}_{MLE}$	${\overset{ˆ}{\beta }}_{MLE}$	${\overset{ˆ}{\gamma }}_{MLE}$
NBPF-Weibull	0.162	0.227	0.411	-
Weibull	0.805	0.769	-	-
F-Weibull	0.260	0.208	-	-
EF-Weibull	0.223	0.215	-	1.170
E-Weibull	0.334	2.684	-	7.470

Using Data 2, Table 7 provides the IC values of the NBPF-Weibull distribution and its related competing distributions. For the NBPF-Weibull model, we have AIC = 53.830, CAIC = 55.094, BIC = 57.237, and HQIC = 54.687; see Table 7. Thus, Table 7 clearly affirms that the NBPF-Weibull distribution has the lowest IC values for Data 2. Therefore, it is apparent that the NBPF-Weibull distribution can be ranked as the $1^{st}$ as it provides a closer fit to Data 2.

**Table 7:** The criterion values of the fitted models using Data 2.

Model	AIC	CAIC	BIC	HQIC
NBPF-Weibull	53.830	55.094	57.237	54.687
Weibull	69.028	69.628	71.299	69.599
F-Weibull	64.767	65.367	67.038	65.338
EF-Weibull	66.677	67.9401	70.083	67.534
E-Weibull	69.681	70.944	73.087	70.537

**Figure 13:**
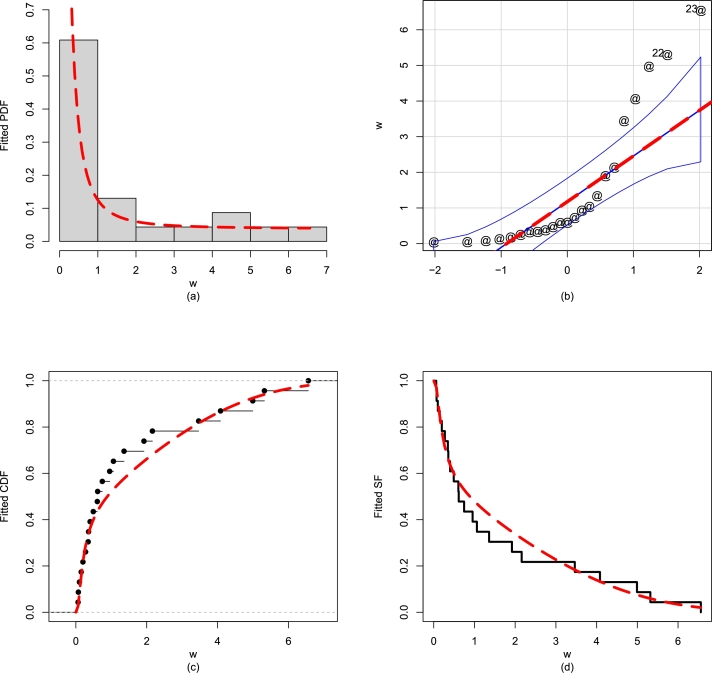
For Data 2, the illustrations of the (a) fitted PDF, (b) QQ plot, (c) empirical CDF, and (d) fitted SF of the NBPF-Weibull distribution.

To support the given illustration in Table 7, we also visually compare and classify the fitted models. For visually illustrating the fitted distributions, we select the QQ, fitted PDF, SF, PP, and empirical CDF; see Fig. 13(a-d). The plots in Fig. 13(a-d) show that the graphical checking tools also support the close fitting ability of the NBPF-Weibull distribution.

### 4.6 Analysis of Data 3

Here, we show the performances of the applied distributions using data on the failure times of electronic machines (i.e., Data 3). We fit the NBPF-Weibull along with competing distributions to Data 3. The values of ${\overset{ˆ}{\alpha }}_{MLE},{\overset{ˆ}{\delta }}_{MLE},{\overset{ˆ}{\beta }}_{MLE}$, and ${\overset{ˆ}{\gamma }}_{MLE}$ are given in Table 8.

**Table 8:** For Data 3, the values of ${\overset{ˆ}{\alpha }}_{MLE},{\overset{ˆ}{\delta }}_{MLE},{\overset{ˆ}{\beta }}_{MLE}$, and ${\overset{ˆ}{\gamma }}_{MLE}$.

Model	${\overset{ˆ}{\alpha }}_{MLE}$	${\overset{ˆ}{\delta }}_{MLE}$	${\overset{ˆ}{\beta }}_{MLE}$	${\overset{ˆ}{\gamma }}_{MLE}$
NBPF-Weibull	0.101	0.106	0.394	-
Weibull	0.662	0.536	-	-
F-Weibull	0.181	0.097	-	-
EF-Weibull	0.107	0.108	-	1.713
E-Weibull	0.616	0.625	-	1.128

**Figure 14:**
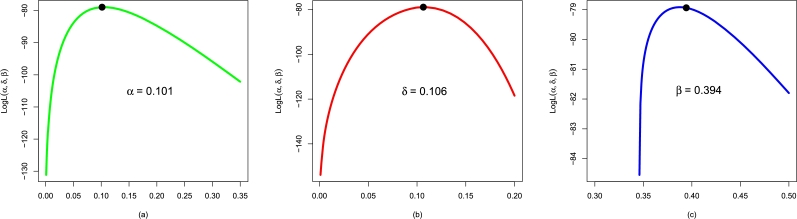
Using Data 3, the profiles plots of (a) ${\overset{ˆ}{\alpha }}_{MLE}$, (b) ${\overset{ˆ}{\delta }}_{MLE}$, and (c) ${\overset{ˆ}{\beta }}_{MLE}$ of the NBPF-Weibull distribution.

Furthermore, Fig. 14(a-c) illustrates the plots for the profiles of ${\overset{ˆ}{\alpha }}_{MLE}$, ${\overset{ˆ}{\delta }}_{MLE}$, and ${\overset{ˆ}{\beta }}_{MLE}$ of the NBPF-Weibull distribution.

Furthermore, the values of the IC for the fitted models are obtained in Table 9. The given results/facts in Table 7 again categorically confirm that the NBPF-Weibull model has the lowest values for Data 3. Using Data 3, for the NBPF-Weibull distribution, we AIC = 163.882, CAIC = 164.404, BIC = 169.619, and HQIC = 166.067. These facts confirm that the NBPF-Weibull distribution is the most optimal and appropriate model for Data 3.

**Table 9:** The criterion values of the fitted models using Data 3.

Model	AIC	CAIC	BIC	HQIC
NBPF-Weibull	163.882	164.404	169.619	166.067
Weibull	208.730	208.986	212.554	210.186
F-Weibull	195.851	196.107	199.675	197.307
EF-Weibull	193.887	194.409	199.623	196.072
E-Weibull	210.750	211.271	216.486	212.934

**Figure 15:**
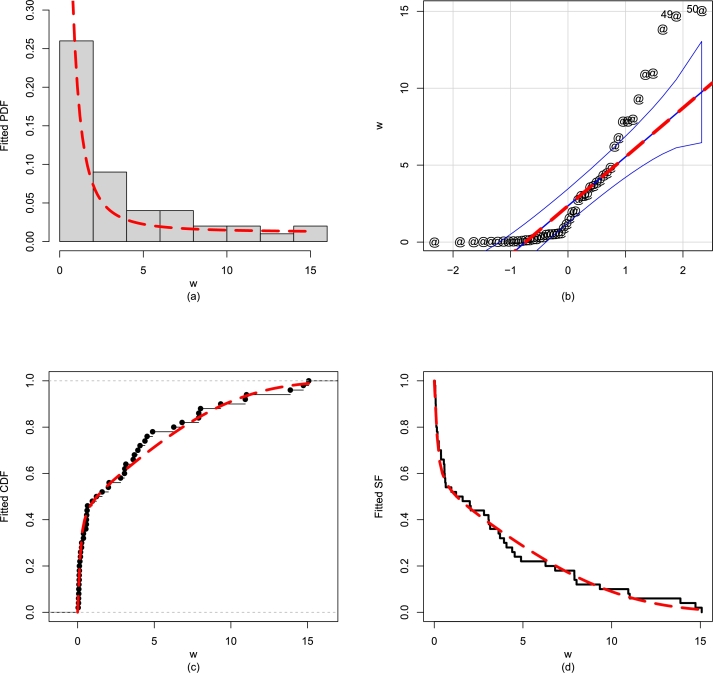
For Data 3, the illustrations of the (a) fitted PDF, (b) QQ plot, (c) empirical CDF, and (d) fitted SF of the NBPF-Weibull distribution.

Besides the numerical comparison of the fitted models, we again show the performances of the NBPF-Weibull model visually. The visual results in Fig. 15(a-d) also show that Data 3 is closely fitted by the NBPF-Weibull distribution.

## 5 Final remarks

This study explored a new extension of the F-Weibull distribution. The new extension of the F-Weibull model was named a NBPF-Weibull distribution. Among certain desirable properties, the NBPF-Weibull distribution produces different patterns of HF including increasing, uni-model, and modified uni-model. Whereas, the PDF of the NBPF-Weibull distribution was capable of capturing right-skewed, symmetrical, and left-skewed patterns. The MLEs of the NBPF-Weibull distribution were mathematically derived. Simulation studies were also carried out. The applicability of the NBPF-Weibull distribution was established by applying it to analyze three practical applications. The IC values were repeatedly supported the superiority of the NBPF-Weibull model

As we have shown, the NBPF-Weibull distribution can capture different patterns of PDF and HF. Furthermore, we also proved its applicability through two practical examples. Despite these virtues, the NBPF-Weibull distribution also has some deficiencies/limitations. For instance, the NBPF-Weibull distribution may not provide the best fit for bimodal data sets, because its PDF does not have a bimodal shape. Moreover, it is also not able to apply the discrete data sets as its continuous model. In the future, one can work further carry this research work to addresses the said demerits of the NBPF-Weibull distribution

However, our intentions regarding future research studies or covering the existing research gaps, including the

• development of the bivariate modification of the NBPF-Weibull distribution that is given by Eq. (5),
• introduction of the discrete modification of Eq. (5),
• employment of the multivariate modification of Eq. (5),
• implementation of the NBPF-Weibull distribution for modeling medical data,
• application of the NBPF-Weibull distribution for taking care of the bivariate data such as (a) import and export, (b) income and expanses, (c) height and weight, (d) distance traveled and the corresponding time consumption, and (e) advertising expanses and sale, among others.
• application of the NBPF-Weibull distribution to hydrological data, and
• Bayesian analysis based on the NBPF-Weibull distribution.

## CRediT authorship contribution statement

**Xiangming Tang:** Writing – review & editing, Writing – original draft, Visualization, Validation, Software, Resources, Methodology, Investigation, Formal analysis, Data curation, Conceptualization. **Jin-Taek Seong:** Writing – review & editing, Writing – original draft, Visualization, Validation, Software, Resources, Methodology, Investigation, Formal analysis, Data curation, Conceptualization. **Randa Alharbi:** Writing – review & editing, Writing – original draft, Visualization, Validation, Software, Resources, Methodology, Investigation, Formal analysis, Data curation, Conceptualization. **Aned Al Mutairi:** Writing – original draft, Visualization, Validation, Software, Resources, Methodology, Investigation, Formal analysis, Data curation, Conceptualization. **Said G. Nasr:** Writing – review & editing, Writing – original draft, Visualization, Validation, Software, Resources, Methodology, Investigation, Formal analysis, Data curation, Conceptualization.

## Declaration of Competing Interest

The authors declare no conflict of interest.

## Data Availability

Data included in article/supplementary material/referenced in article.
